# Extensive Sampling of Molecular Dynamics Simulations to Identify Reliable Protein Structures for Optimized Virtual Screening Studies: The Case of the hTRPM8 Channel

**DOI:** 10.3390/ijms23147558

**Published:** 2022-07-08

**Authors:** Silvia Gervasoni, Carmine Talarico, Candida Manelfi, Alessandro Pedretti, Giulio Vistoli, Andrea R. Beccari

**Affiliations:** 1 Dipartimento di Scienze Farmaceutiche, Università degli Studi di Milano, Via Luigi Mangiagalli, 25, I-20133 Milano, Italy; silgervasoni@gmail.com (S.G.); alessandro.pedretti@unimi.it (A.P.); giulio.vistoli@unimi.it (G.V.); 2 Dompé Farmaceutici SpA, EXSCALATE Labs, Via Tommaso De Amicis, 95, I-80131 Napoli, Italy; carmine.talarico@dompe.com (C.T.); candida.manelfi@dompe.com (C.M.)

**Keywords:** virtual screening, systematic sampling, MD simulations, consensus models, LiGen software, EFO algorithm, hTRPM8

## Abstract

(1) Background: Virtual screening campaigns require target structures in which the pockets are properly arranged for binding. Without these, MD simulations can be used to relax the available target structures, optimizing the fine architecture of their binding sites. Among the generated frames, the best structures can be selected based on available experimental data. Without experimental templates, the MD trajectories can be filtered by energy-based criteria or sampled by systematic analyses. (2) Methods: A blind and methodical analysis was performed on the already reported MD run of the hTRPM8 tetrameric structures; a total of 50 frames underwent docking simulations by using a set of 1000 ligands including 20 known hTRPM8 modulators. Docking runs were performed by LiGen program and involved the frames as they are and after optimization by SCRWL4.0. For each frame, all four monomers were considered. Predictive models were developed by the EFO algorithm based on the sole primary LiGen scores. (3) Results: On average, the MD simulation progressively enhances the performance of the extracted frames, and the optimized structures perform better than the non-optimized frames (EF1% mean: 21.38 vs. 23.29). There is an overall correlation between performances and volumes of the explored pockets and the combination of the best performing frames allows to develop highly performing consensus models (EF1% = 49.83). (4) Conclusions: The systematic sampling of the entire MD run provides performances roughly comparable with those previously reached by using rationally selected frames. The proposed strategy appears to be helpful when the lack of experimental data does not allow an easy selection of the optimal structures for docking simulations. Overall, the reported docking results confirm the relevance of simulating all the monomers of an oligomer structure and emphasize the efficacy of the SCRWL4.0 method to optimize the protein structures for docking calculations.

## 1. Introduction

Structure-based virtual screening (VS) simulations comprise a set of well-established in silico approaches which proved successful in hit identification and drug repurposing [1,2]. While involving various computational protocols, they are unified by the common pivotal role played by docking simulations [3]. Consequently, the availability of reliable protein structures is a mandatory prerequisite to perform successful analyses [4]. Such a requirement might be fulfilled when protein structures in complex with suitable ligands have been experimentally resolved. Indeed, these structures should assure both a satisfactory structural reliability and a properly arranged binding site [5]. In contrast, the resolved structures in their unbound state as well as almost all theoretical models, even when assuring the necessary structural quality, pose the problem regarding the correct arrangement of their binding pockets [6,7].

A strategy usually adopted in these cases involves rather long preliminary MD simulations by which the protein structures should be reasonably optimized with beneficial effects on the architecture of their binding sites [8]. Even after discarding redundant frames, the performed MD runs generate a high number of representative protein conformations and the selection of the optimal structure(s) for the following docking simulations represents a crucial step to maximize the predictive performance of the resulting VS campaigns [9].

Therefore, one may imagine two typical situations. In the first case, useful information concerning the precise architecture of the binding pocket can be derived by resolved homologous proteins or by literature data. In this fortunate condition, docking simulations can be focused on those MD frames in which at least the binding pocket is in best agreement with the experimental references [10]. In the second unfortunate situation, experimental data regarding the arrangement of the binding pocket are not available and consequently the selection of few optimal frames must be replaced by a rational analysis of the entire MD trajectory [11]. To this end, different approaches have been proposed ranging from a simple sampling of the entire trajectory to a rational selection of the most representative frames based on essential dynamics or other energy-based criteria [12].

In a previous study, we experienced the first situation to develop targeted protocols for optimized VS campaigns on the hTRPM8 structure [13]. Along with its physiological role and medicinal interest [14], the TRPM8 ion-channel was selected due to the availability of resolved structures (from *ficedula albicollis* and *parvus major*) both in the apo state and in complex with known modulators [15]. Due to the high conservation degree, these experimental structures can be conveniently used to build reliable homology models for the hTRPM8 structure in its homotetrameric state. Hence, a hTRPM8 homology model was generated by using the corresponding apo state from *ficedula albicollis* as the template [16] and the obtained structure underwent a reasonably long MD run (1.25 μs). This simulation had the objective to equilibrate the hTRPM8 theoretical model as well as to provide a set of representative conformations to be used in the following docking simulations. In this fortunate case, the other available co-crystallized TRPM8 structures can be utilized as the templates to identify the best frames for docking simulations. By combining structural comparison and re-docking experiments, a representative set of few satisfactory hTRPM8 structures were selected. The resulting VS campaigns proved successful in reaching very remarkable predictive performance and emphasized the relevance of repeating docking calculations on all four monomers of the selected hTRPM8 conformations.

In the present study, we put ourselves in the second unfortunate situation by pretending not to have information about the architecture of the hTRPM8 binding cavity. Stated differently and instead of focusing the VS campaigns on few properly selected suitable frames, we performed an extended set of docking simulations by systematically sampling the entire MD trajectory. Thus, the primary objective of this study is to assess whether such a blind and methodical strategy can be effective by comparing the here obtained performances with those reached in the previous study [13]. Clearly, this comparative study is targeted for the hTRPM8 protein, but the proposed computational procedure could be fruitfully applied in all docking studies when experimental data on the binding site(s) are not yet available.

## 2. Results

### 2.1. Docking Simulations

As mentioned in the Introduction, the study involved an extended set of VS simulations performed by exploiting the already published MD simulation (1.25 μs) of the hTRPM8 homology model in its homotetrameric assembly and in its apo state [13]. In detail, such a systematic study comprised 50 docking runs by simulating a memorized frame every 25 ns. To speed up such an extensive set of docking simulations, a subset of the database utilized in the previous study was collected. Such a subset was composed by 1000 molecules comprising 20 known TRPM8 inhibitors and 980 inactive decoys. For the same reason, the docking simulations were carried out using only the LiGen program [17] which reached the best performance in the previous study (compared to PLANTS and GOLD). Similarly, the performed predictive analyses involved only the primary scores computed by the LiGen program without rescoring calculations.

Notwithstanding the above, the docking simulations involved all the four monomers of all the 50 considered frames since this exhaustive exploration played a noteworthy enhancing role in the previous study [13]. Furthermore, the previous docking experiments were unable to provide convincing results about the beneficial effect of energy minimization of the selected protein structures; therefore, a completely new approach was tested here. This is based on the SCWRL4 software [18] which adds the side-chains to a protein structure by an algorithm which selects them from a rotamer library by a backbone-dependent approach. Such a selection also accounts for the interaction potential elicited by each side-chain to find the rotamer that best fits its protein micro-environment. Hence, all docking simulations were repeated by considering the frames as extracted from MD trajectory and after the side-chain optimization performed by SCWRL4. Finally, and to better explore the role of multiple binding modes, 10 poses were generated for each ligand and the corresponding best and average score values were used during the predictive analyses according to the concept of the binding space [19].

The so computed docking scores were utilized to develop consensus models by using the Enrichment Factor Optimization (EFO) algorithm [20,21]. This linearly combines docking scores by maximizing a quality function primarily based on the resulting EF 1% values. For each analysis, the consensus equations were generated by including at most three variables by using a recently proposed EFO release which implements an incremental method and stops the search if the inclusion of an additional variable does not improve the resulting EF1% values [22].

### 2.2. VS Campaigns by Using the Non-Optimized Frames

As a preamble, it should be noted that here and in the following analyses (see Section 2.3), the docking results of a given monomer were discarded when LiGen was unable to properly accommodate more than three active molecules. A frame was entirely discarded when more than two monomers showed unsuitable docking results.

Table 1 compiles the best consensus linear equations with the corresponding EF1% values for the frames which fulfilled the above-described criteria. Interestingly, the first three frames (t = 25, 50, and 75 ns) were discarded while all the following 47 frames were accepted. This finding suggests that the first relevant effect induced by the MD simulation is the optimization of the overall arrangement of four binding cavities. They may be slightly constrained in the starting structures and become increasingly more relaxed during the simulation and in particular after about 100 ns. The beneficial effect exerted by the MD run is further evidenced by considering that in all the first 13 frames (until 275 ns) at least 1 monomer does not fulfill the defined criterion and overall 21 monomers out of 52 were discarded (data not shown). In contrast, only 8 monomers (out of 148) were discarded in the following 37 frames (925 ns). In more detail, the monomer D is the most frequently discarded one (17 out of 29), followed by monomer A (9 cases), while monomers B (3 cases) and C (0 cases) were virtually never removed.

**Figure 1:**
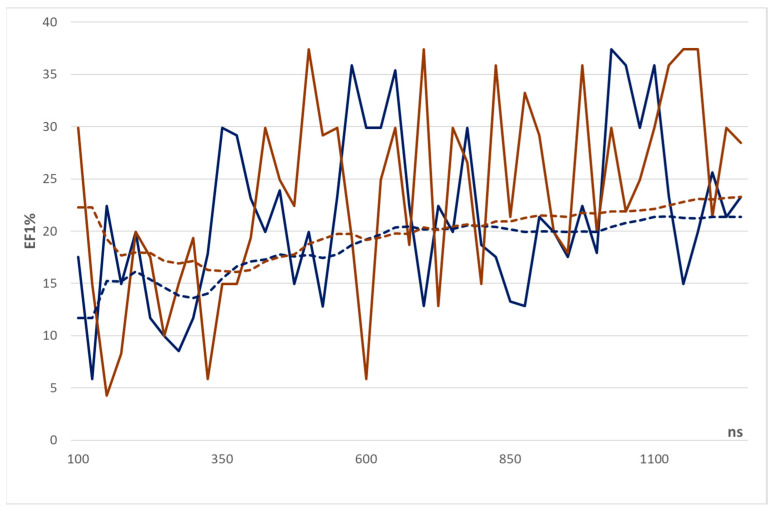
Trends of the EF1% values (bold lines) and the EF1% cumulative means (dashed lines) for non-optimized (blue lines) and optimized (brown lines) frames as extracted from the MD run.

The beneficial effect exerted by the MD simulation on the architecture of the binding cavities is also confirmed by the overall increase in the corresponding EF1% values during the MD run as seen in Table 1 and Figure 1. The trend of the EF1% values and the comparison with that derived by using the optimized frames will be further discussed in Section 2.4. While evidencing an overall increasing trend, Table 1 reveals that the EF1% values retain a marked variability with a persistent up and down profile with EF1% values which ranges from 5.84 to 37.38. On one hand, such a variability emphasizes the key role of the performed MD run on the hTRPM8 tetramer to reach convenient arrangements of its binding sites. On the other hand, this underlines the marked flexibility of the explored cavities and suggests that even the fluctuations of few side chains can influence the reliability of the docking simulations. As discussed in the previous study, such a flexibility can also be amplified by the dynamic cross-talk between the interacting monomers [13]. This can explain why the involvement of all four monomers in these VS campaigns exerts a beneficial effect on the resulting predictive models.

While avoiding a detailed analysis of the compiled consensus models, Table 2 reports the relevance of the four monomers in the best equations and reveals a significant difference compared to the previous study [13]. Indeed, when using few rationally selected protein conformations, docking results unraveled a well-defined trend with the monomer A playing a prevailing role in determining the predictive performance. By contrast, when repeating the docking simulations on an extended set of frames, a different behavior is observed. The monomers B and C are the most frequent ones, the monomer A plays an in-between role, while the monomer D is rarely included in the selected models. The monomer D is also the most frequently discarded subunit (see above) and this suggests that its binding cavity has an intrinsic difficulty in assuming arrangements suitable for binding.

Table 2 highlights that best and mean score values show a similar incidence. Nevertheless, Table 1 reveals that the frequency of mean scores increases during the MD simulation. Indeed, the number of mean scores in the second half of the MD run (0.625 to 1.25 μs) is markedly higher compared to the first half (34 vs. 21). This confirms that the binding cavities are characterized by a rather constrained arrangement at the beginning of the simulation. These constraints hamper the mobility of the docked ligands and minimizes the enhancing effect of accounting for different poses. In contrast and during the MD run, the binding pockets show increasingly relaxed and wider arrangements. This permits a greater mobility of the bound ligands which can assume different binding modes and explains the increasing role of the mean scores. Finally, the analysis of the frequency of the three LiGen primary scores reveals that CS and CSopt scores have similar relevance (55 and 42 occurrences for CS and CSopt, respectively), while PS score is virtually never included (3 occurrences).

### 2.3. VS Campaigns by Using the SCRWL4 Optimized Frames

Similarly to Table 1, Table 3 includes the best models and the relative EF1% value for the 50 optimized frames. Table 3 shows that there is not a clear match between the frames discarded in the two sets of simulations, although the criteria for discarding monomers and entire frames are the same as described above. In detail, Table 3 highlights that there are three discarded frames as seen in Table 1, even though they are not focused on the beginning of the MD simulation (as seen before) but are distributed throughout the entire simulation. The analysis of the discarded monomers (data not reported) showed that the total number of discarded monomers is here lower than that seen with non-optimized frames (21 vs. 29). Moreover, they are less focused on the first part of the MD run (11 in the first 275 ns and 10 in the following 925 ns) and this can explain the different distribution of the discarded frames. The frequency with which the four monomers are discarded is similar to that previously seen with monomers A and D being frequently removed (with 11 and 8 cases, respectively), while monomers B and C are almost never discarded (with 0 and 2 cases, respectively). Taken together, the analysis of the discarded monomers suggests that the optimization by SCRWL4 is unable to completely upset the reliability of the simulated frames (especially because this does not alter the protein backbone) even though this approach is able to induce an overall structural enhancement which appears particularly relevant for the first part of the MD run.

Even though the detailed comparison of the two sets of docking simulations will be discussed in the next section, a rapid analysis of Table 3 confirms that the EF1% values also increase here during the MD run. More importantly, the VS campaigns performed by using the optimized frames perform better than those carried out by non-optimized structures. The better performance of the optimized frames is witnessed by both the average EF1% values (21.38 vs. 23.29) and the number of frames which show noteworthy EF1% values (i.e., >30, 7 vs. 5). As previously seen, the best models collected by Table 3 show a persistent up and down profile in their performances (EF1% values from 5.84 to 37.88) and include a variable number of parameters.

Table 4 compiles the frequency with which the monomers appear in the best equations collected by Table 3 and reveals some key differences compared to the non-optimized frames (see Table 2). In detail, the monomer B is the most involved one followed by the monomer C, while the monomers A and D play more limited roles. The mean scores are here almost double the best values, a ratio already seen for the last non-minimized frames (see Table 1). These results indicate that the optimization by SCRWL4.0 is able to reduce the constraints of all the simulated frames. This increases the wideness of their binding sites; thus, promoting the ligand capacity to assume multiple binding modes as encoded by the average scores. This finding is clearly confirmed by the volume averages reported in Table 2 and Table 4: the optimized binding sites show a volume increase of 39% (from 407 to 654 Å^3^). Regarding the frequency of the various primary LiGen scores, Table 3 shows results superimposable to those of Table 1 since the CS and CSopt scores reveal prevailing and comparable roles (41 and 52 occurrences, respectively) while the PS score appears only 5 times.

### 2.4. Comparison of the Two Sets of Simulations

Figure 1 compares the performances of the two sets of docking simulations as described by the best EF1% values and by the EF1% cumulative means. Except for the high EF1% value reached by the optimized frame at 100 ns, both sets of calculations reveal an increasing trend in their EF1% values which can be better evidenced when considering the cumulative EF1% means. The cumulative trends also offer a clear confirmation of the better performance reached by the optimized frames and reveal that such a superiority is already evident at the beginning of the MD run and remains roughly constant throughout the simulation with differences in cumulative EF1% means around 2.0. A more detailed analysis of Figure 1 highlights that the two trends show a similar behavior and in both cases the largest EF1% enhancements are seen between 400 and 800 ns. This observation suggests that the MD run can be subdivided into three segments.

The first equilibration part, which roughly involves the first 400 ns, is characterized by the optimization of the overall folding of the simulated homotetramer. As previously reported, this process is driven by a reinforcement of inter-monomeric interactions [13]. This process has a limited impact on the fine architecture of the binding pockets which retain the constraints characterizing the starting structures. Hence, the VS performances of the corresponding frames do not significantly increase during this first phase. This first part shows marked differences in the performances reached by optimized and non-optimized frames. This confirms that the largest enhancing effect played by the SCRWL method is focused on these first frames. The second relaxation phase, which comprises the frames between 400 and 800 ns, involves a further enhancement of the overall quaternary hTRPM8 structure but also a beneficial relaxing of the explored binding sites which increase their flexibility; thus, promoting the ligand recognition. This is reflected in the increase in the resulting performances as encoded by their EF1% values. The third stabilization phase, which roughly corresponds to the last 400 ns, is characterized by the tetrameric hTRPM8 structure which has reached a reasonable equilibrium and shows, at most, minor conformational fluctuations with limited effects on the predictive performances.

Such a subdivision scheme is partly confirmed by Table 5 which reports the intermediate EF1% means values for both sets of VS campaigns as computed for segments of 250 ns. The optimized frames exhibit a clear increasing trend in the EF1% values during the MD run, while the non-optimized structures retain a more marked up-and-down profile as evidenced by the drop in EF1% mean between 775 and 1000 ns. When focusing on the first 750 ns, the largest difference between optimized and non-optimized frames is seen in the first 250 ns. This finding confirms that the here applied optimization procedure is able to reduce the constraints that affect the starting structures with positive effects on the interaction capacity of their binding pockets (as discussed above). The beneficial effects of the above-described relaxation phase is here documented by considering that the significant EF1% increases in both VS sets are seen between the 275–500 and 500–750 periods. In the second and third segments, the EF1% means between the two sets are similar (ΔEF1% less than 1.0). This indicates that the optimization process by SCRWL4.0 cannot further improve the frames already relaxed by the MD simulation. The last part of the MD run highlights less coherent performances due to the already mentioned drop for the non-optimized frames. As already noticed in Figure 1, Table 5 confirms that the overall EF1% difference between the two sets of docking simulations is around 2.0.

### 2.5. Multiple Frames Consensus Strategy

While avoiding time-demanding rescoring calculations, the last analyses of the study involved a consensus strategy in which the primary scores of some selected highly performing frames were combined. This strategy was applied to both non-optimized and optimized structures by focusing on the frames with EF1% > 30 (5 and 7 for non-optimized and optimized frames, respectively). Table 6 compiles the results obtained by these analyses and reveals that the consensus approach based on multiple frames proved successful in enhancing the performances for both non-optimized and optimized structures. In detail, the best performances are reached by combining the optimized frames which yield the best EF1% values as well as the highest EF1% means (as derived by averaging the EF1% of the best 20 generated models). The difference between non-optimized and optimized frames is in agreement with what was already observed for the single frames (around 2.0) and the same difference is also seen in mean values. Concerning the specific role of the four monomers, Table 6 indicates that monomers C and D play key roles for all frames, while monomers A and B reveal less constant roles. Finally, Table 6 confirms the major role of mean scores in both analyses. The obtained models were further assessed by y-scrambling and the obtained average EF1% values were equal to 18.93 and 10.86 with all monomers for non-optimized and optimized frames, respectively. Such a decrease in performance indicates that these models are unlikely to be biased by chance.

The analysis of the occurrence of the selected frames in the best 20 models as generated by considering all monomers reveals that some frames have a very high frequency (data not shown). Specifically, optimized frames 1125 and 1175 have a predominant role and similarly non-optimized frames 650 and 1025 are highly frequent in the corresponding models. This finding suggests that productive models might be developed even combining a more limited number of frames compared to what was reported in Table 6.

### 2.6. Structural Analysis of the Sampled Frames

Based on the obtained results, the already reported MD trajectory was re-analyzed to highlight structural features of the selected frames which can be related to their predictive performance. Since the main effect exerted by the MD run is a general relaxation/equilibration of the overall hTRPM8 tetramer which reduces the structural constraints also affecting the binding cavities, this analysis was primarily focused on the volume of the binding sites as computed by POVME [23].

**Figure 2:**
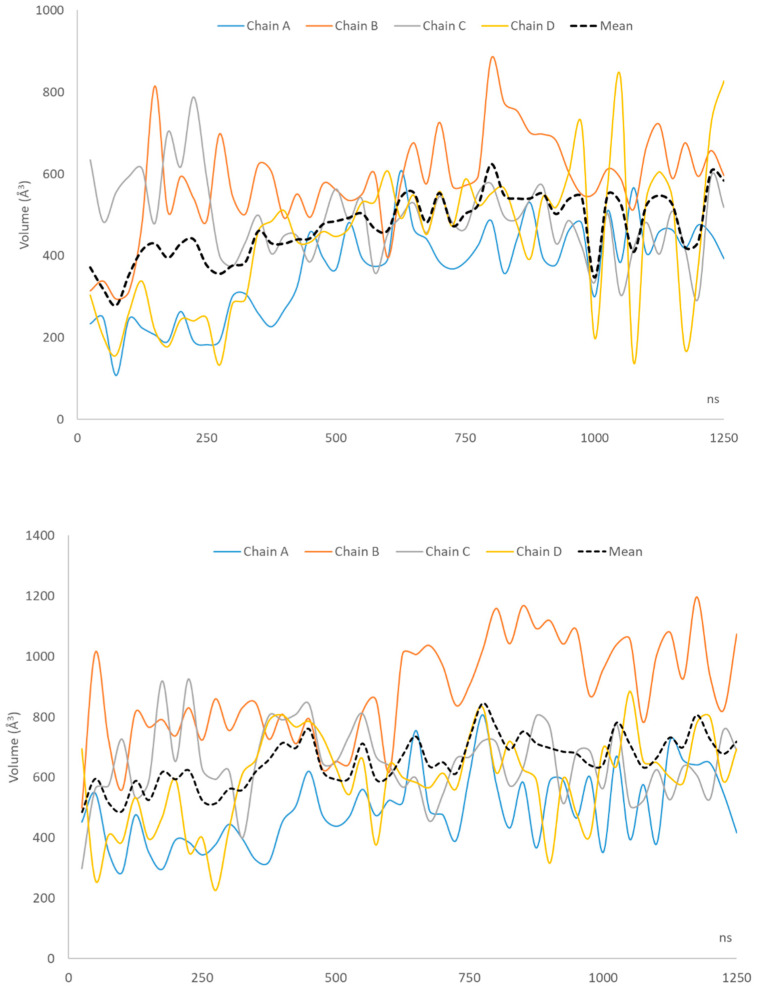
Profiles of the volumes of the four binding pockets within non-optimized (**top panel**) and optimized (**bottom panel**).

Figure 2 shows how the volume of the binding pockets varies during the MD run by considering the four subunits of the sampled frames without and after SCRWL-based optimization. The corresponding volume values can be found in Table S1, while the average values are compiled in Table 5. The mean volumes for the four monomers are compiled in Table 2 and Table 4. The first consideration involves the average values which are significantly greater in the optimized frames for all four subunits. This confirms that the SCRWL procedure is able to relax and to expand the binding cavities even without affecting the backbone conformation. While showing persistent up and down trends, the four monomers reveal similar profiles in the two plots which allow two different behaviors to be identified. Indeed, monomers A and D show increasing volumes during the MD simulation and this is more evident in the non-optimized frames where a sharp increase is observed around 400 ns. In contrast, monomers B and C reveal rather constant volume values for their binding pockets without significant differences between optimized and non-optimized structures.

There is no correlation between the trends of the four subunits nor between the volume profiles of the optimized and non-optimized frames. The only weak correlation (r^2^ = 0.43) can be seen in the trends of the volumes averages of the four cavities, which confirm that (1) the binding pockets experience an overall widening during the MD run and (2) the overall cavities of the optimized frames are constantly and significantly greater than those of the non-optimized structures.

To further investigate the role of the computed volumes in determining the reached performances, the correlations between volumes of the binding sites and predictive performances were analyzed. While there are no significant relations between EF1% and volume values by considering all the analyzed frames, significant correlations can be found between volume and EF1% averages as reported in Table 5 with the optimized frames which afford a better relationship than the non-optimized ones (r = 0.94 vs. 0.73). Furthermore, contrasting results are derived when correlating the volume averages with the frequencies with which the four monomers are involved in the predictive models (as reported in Table 2 and Table 4). There is indeed a very remarkable correlation for the optimized frames (r = 0.99) while the non-optimized ones provide a poor relation (r = 0.55) mostly ascribable to the outlier behavior of monomer A.

Taken together, the analysis of the volumes of the binding pocket emphasizes the key role of the pocket size in determining the performance of each frame. Such a role can find two different explanations. On one hand, one may figure out that the wider the pocket, the easier the ligand finds a convenient pose. On the other hand, one may hypothesize that a wide binding pocket is also suggestive of a well-relaxed structure which reached an optimal arrangement for the ligand recognition.

## 3. Discussion

As stated in the Introduction, the primary objective of the study was to assess if a methodical sampling of the frames generated by MD runs can be effective in virtual screening campaigns when experimental data to guide the selection of few optimal structures for docking are not available. Such an analysis can be performed by comparing the here reached performances with the previously published results involving few optimal hTRPM8 structures chosen based on the available experimental data and structures [13]. Even though the different composition of the two screened datasets and in particular the different abundance of active molecules (here 2%, namely 20/1000 vs. 1% in the previous study, namely 53/5300) prevents an easy comparison of the reached performances, the metrics reported in Table 6 allow for some insightful considerations.

The EF1% values are clearly affected by the above-mentioned differences in the dataset composition. Nevertheless, Table 7 reveals that the EF1% value reached by the here developed best models correspond to a high percentage of active molecules within the top 1%. This means that these models proved satisfactory at least in the early recognition feature. Regarding the overall metrics, Table 7 shows that the previous best model performs slightly better than those presented here with the optimized frames which yield better performances. Based on these results, the consensus approach based on multiple frames appears a very promising strategy since it does not require additional calculations and allows a precise evaluation of the role of the best performing frames. Thus, it can guide the rational selection of the frames on which the following docking calculations can be focused.

Clearly, the richness of structural information generated by a rather extended MD simulation can be exploited by various computational strategies. Thus, one may imagine more systematic and combinatorial approaches which are based on the consideration that each ligand should prefer a specific frame or more compact approaches in which all the generated frames are reduced to few representative structures as derived by clustering and averaging methods or different energy-based prioritization. This study was designed by assuming that a reliable target structure for docking simulations cannot be optimal for all simulated ligands (this cannot happen even using resolved structures) but it should represent a good compromise able to afford reliable complexes for most ligands. In order to find these reliable structures, this study was designed to reach an optimal balance between exhaustiveness and speed of the calculations. The here obtained results indicate that the systematic sampling of a given MD trajectory can provide VS performance in substantial agreement with that reached by using few rationally selected structures.

## 4. Methods and Materials

### 4.1. Frame Selection and Optimization

As mentioned above, the frames were extracted from the MD run of 1.25 μs involving the hTRPM8 in its tetrameric assembly and already reported in a previous study [13]. In detail, the present study considered 50 frames as obtained by systematically sampling the MD trajectory and extracting a frame every 25 ns. For each sampled frame, the following docking simulations involved the entire homotetramer by considering all four binding pockets. In more detail, docking simulations were performed by considering the frames as directly extracted from the MD trajectory as well as after optimization by using SCRWL4.0 [18]. Notice that this software was primarily developed to add the optimal side-chain rotamers during the generation of theoretical models. Here, a different application for SCRWL4.0 is proposed that is the optimization of the frames extracted from a MD simulation by enhancing the conformational profiles of the sole side-chains. Stated differently, such an optimization procedure does not perturb the backbone folding but improves the arrangement of the side-chains in order to optimize the fine architecture of the binding pockets. Hence, the SCRWL4.0 tools was used to optimize all the 50 extracted frames by applying the default parameters. Finally, the volume of the binding cavities of the simulated frames was calculated by using POVME [23].

### 4.2. Virtual Screening by Using LiGen

Docking simulations involved a randomly extracted subset of the dataset already utilized in previous studies, which was composed of 5300 molecules, 53 of which are known hTRPM8 modulators and 5247 are experimentally proven as non-binders [24]. In detail, the here utilized subset comprises 1000 molecules among which 20 are active ligands. Each ligand was prepared by considering the predominant form at physiological pH as previously described [24]. Docking simulations were performed using LiGen and were focused on 10 Å radius sphere around the center of mass described by the residues Tyr745, Asn799, Asp802, and Tyr1005 which play a well-known role in ligand recognition [25]. The geometrical docking procedure implemented in LiGen, which follows a specific workflow to compute three docking scores, was used for the docking simulations. First, the Pacman Score (PS) estimates a geometric fitting score to evaluate the interaction between a ligand conformation and the pocket, basing on shape and volume information; then, the Chemical Score (CS), representing the ligand binding energy, is calculated by using an in-house developed scoring function. Lastly, a minimization algorithm that treats the docket ligand as a rigid body inside the binding site, called the Optimized Chemical Score (Csopt) is evaluated [17].

### 4.3. Rescoring and Consensus Analyses

For all the sampled frames (both optimized and non-optimized) and for all the four simulated monomers, the primary scores as generated by LiGen were utilized to develop consensus equations by using the EFO algorithm [20,21]. This linearly combines the input scores by optimizing a search function based on both the resulting EF1% value for early recognition and a skewness function which accounts for the distribution of the active compounds in the entire ranking. In detail, these analyses involved the generation of consensus models including at most three variables by applying an incremental version of the EFO algorithm which stops the model generation if the inclusion of an additional variable does not enhance the resulting performances [22]. For each ligand and each scoring function, the input variables comprise the best and the mean values as obtained by averaging the 10 generated poses using the VEGA suite of programs [26]. The predictive power of the developed models was assessed by subdividing the considered dataset in training (70%) and test (30%) sets and this validation was repeated 5 times to minimize the randomness. The validation phase was utilized to prioritize the generated models and to compute the reported EF1% values. The consensus models from multiple frames were also assessed by y-scrambling as implemented by the EFO tool.

## 5. Conclusions

Although the reported results should be confirmed by additional studies involving different targets and/or various sampling procedures, the present study emphasizes the efficacy to combine MD simulations with docking calculations to improve the predictive power of the obtained results and reveals how a systematic sampling can be a suitable strategy when experimental data does not permit a precise selection of the optimal target structures. Finally, the obtained results confirm the efficacy to simultaneously consider all the subunits of an oligomeric target for docking simulations and reveal the beneficial role of the SCRWL4 method to optimize the fine arrangement of the explored binding sites.

## Figures and Tables

**Table 1:** Best predictive consensus models and relative EF1% values as obtained by the non-optimized frames.

Time (ns)	EF1%	Consensus Equation
25	nd	The frame was fully discarded
50	nd	The frame was fully discarded
75	nd	The frame was fully discarded
100	17.51	1.00 PSmeanB
125	5.84	1.00 CSbestC
150	22.42	−1.00 CSbestC − 0.36 CsoptbestB
175	14.95	1.00 CsoptmeanC + 2.96 CSbestB
200	19.93	1.00 CSmeanB − 1.10 CSbestB − 1.38 CsoptmeanA
225	11.67	1.00 PSmeanA
250	9.96	−1.00 CSmeanC − 2.44 CSmeanB
275	8.54	−1.00 CSmeanC + 0.46 CSbestB
300	11.67	1.00 CSbestC
325	17.81	1.00 CSbestD − 1.11 CSbestC
350	29.90	−1.00 CsoptbestB + 2.15 CsoptbestA
375	29.18	1.00 CSbestD
400	23.12	−1.00 CSmeanD − 8.80 CsoptbestC
425	19.93	1.00 CsoptmeanB − 3.53 CSmeanA
450	23.92	−1.00 CSmeanD − 0.41 CsoptbestB − 0.11 CSmeanA
475	14.95	−1.00 CSmeanD − 9.86 CsoptbestC + 3.87 CsoptbestA
500	19.93	1.00 CSmeanD − 12.42 CsoptbestA
525	12.81	1.00 PSmeanD − 71.23 CSmeanD − 58.51 CsoptbestC
550	23.35	1.00 CSbestA
575	35.88	−1.00 CsoptmeanC + 1.30 CSbestA
600	29.90	−1.00 CsoptbestC − 0.39 CSmeanA
625	29.90	−1.00 CsoptmeanD − 0.082 CSmeanB + 1.26 CsoptmeanA
650	35.88	−1.00 CSbestD − 1.71 CSmeanC + 1.16 CsoptbestB
675	22.42	−1.00 CsoptmeanC + 0.24 CSbestA
700	12.82	−1.00 CSbestB + 0.82 CSmeanA
725	22.42	−1.00 CSmeanB + 0.36 CSbestB
750	19.93	−1.00 CSmeanC − 6.04 CsoptmeanB − 1.51 CsoptmeanA
775	29.90	−1.00 CSmeanC + 0.67 CsoptbestB
800	18.69	−1.00 CsoptmeanB + 0.24 CSbestA
825	17.51	1.00 CSBestA
850	13.29	1.00 CSbestD + 44.18 CsoptbestC
875	12.82	−1.00 CSoptmeanB + 7.20 CSmeanB − 29.71 CSbestA
900	21.36	−1.00 CSbestC + 0.95 CSbestA
925	19.93	1.00 CsoptmeanD + 4.31 CSmeanC + 0.90 CSbestC
950	17.51	1.00 CSbestC
975	22.42	−1.00 CSmeanD + 0.94 CSbestC
1000	17.94	−1.00 CsoptmeanC + 0.42 CSmeanB − 0.15 CSmeanA
1025	37.38	1.00 CSoptmeanD − 1.27 CsoptbestC
1050	35.88	1.00 CsoptmeanD + 1.69 CSmeanC − 9.10 CsoptmeanB
1075	29.90	1.00 CSbestC − 1.86 CsoptmeanB
1100	35.88	−1.00 CSmeanB − 4.56 CsoptbestB − 3.70 CSmeanA
1125	23.35	1.00 CSbestA
1150	14.95	−1.00 CsoptmeanD − 0.67 CsoptmeanC + 0.34 CSbestB
1175	19.93	−1.00 CSbestB + 0.32 CsoptmeanA
1200	25.63	−1.00 CsoptmeanC − 1.85 CsoptbestB + 0.95 CsoptmeanA
1225	21.35	−1.00 CsoptmeanD + 0.27 CSbestC
1250	23.26	1.00 CsoptmeanC − 10.07 CsoptmeanB + 1.90 CsoptmeanA

**Table 2:** Frequency of the scores from the four simulated non-optimized monomers in the selected consensus equations. The table also includes the mean volumes of the four binding sites in the 50 selected frames (in Å^3^).

Score	Monomer A	Monomer B	Monomer C	Monomer D	Total
Best	11	14	16	4	45
Mean	13	15	14	13	55
Total	24	29	30	17	100
Mean volume	365.4	582.2	492.7	439.8	470.0

**Table 3:** Best predictive consensus models and relative EF1% values as obtained by the selected optimized frames.

Frame	EF1%	Consensus Equation
25	29.18	1.00 PSmeanC
50	11.67	1.00 CSbestB
75	25.63	−1.00 CsoptbestB − 1.27 CsoptmeanA
100	29.90	−1.00 CSbestC + 1.22 CSmeanB − 0.21 CSbestA
125	14.95	−1.00 CsoptmeanD + 0.89 CSmeanD
150	4.27	1.00 CsoptbestC − 1.42 CsoptmeanB
175	8.31	−1.00 CSmeanD + 0.55 CSmeanC + 0.27 CSmeanB
200	19.93	−1.00 CsoptmeanD − 5.44 CSbestA
225	17.51	1.00 CSbestA
250	9.96	−1.00 CSmeanC − 2.44 CSmeanB
275	nd	The frame was fully discarded
300	19.33	1.00 CSmeanD + 2.91 CSbestC − 7.81 CsoptbestB
325	5.84	1.00 CSmeanD
350	14.95	−1.00 CSbestC − 0.70 CsoptbestB
375	14.95	−1.00 CsoptbestC − 1.14 PSmeanA
400	19.33	−1.00 CSmeanC − 4.23 PSmeanB + 18.23 PSbestB
425	29.90	−1.00 CsoptmeanC − 10.07 CSmeanB + 1.95 CsoptbestA
450	24.92	1.00 CsoptmeanD − 3.48 CSmeanA
475	22.42	−1.00 CSmeanD − 21.50 CSmeanC
500	37.38	−1.00 CsoptmeanC + 1.73 CSmeanA
525	29.18	1.00 PSmeanD
550	29.90	−1.00 CsoptmeanC − 8.69 CsoptbestC + 0.27 CSmeanB
575	nd	The frame was fully discarded
600	5.84	1.00 CbestA
625	24.92	1.00 CSbestD − 14.04 CsoptmeanA
650	29.90	1.00 CsoptbestB − 1.66 CSmeanA − 0.94 CsoptbestA
675	18.69	1.00 CsoptbestC − 1.09 CsoptmeanB
700	37.38	1.00 CsoptmeanC − 4.44 CsoptmeanB
725	12.82	−1.00 CSmeanD − 1.38 CsoptbestB
750	29.90	1.00 CSmeanD − 0.12 CsoptbestD − 1.14 CsoptmeanA
775	26.58	1.00 CsoptmeanD − 1.25 CSoptbestC
800	14.95	1.00 CsoptmeanD − 2.89 CsoptmeanA − 9.20 CSbestA
825	35.88	1.00 CsoptmeanD − 1.05 CsoptmeanB
850	21.35	1.00 CsoptmeanC − 3.50 CsoptmeanB
875	33.22	−1.00 CsoptbestD − 0.49 CsoptmeanA
900	29.18	1.00 CSbestA
925	19.93	−1.00 CSbestD − 1.34 CSmeanC − 2.32 CSmeanB
950	17.94	1.00 CsoptbestC − 12.25 CSbestA
975	35.88	−1.00 CsoptmeanD − 0.68 CsoptmeanB
1000	19.93	−1.00 CsoptmeanC − 0.48 CSmeanB − 4.85 CsoptbestB
1025	29.90	−1.00 CsoptmeanB + 0.060 CsoptbestA
1050	Nd	The frame was fully discarded
1075	24.92	−1.00 CsoptbestD − 3.30 CSmeanB
1100	29.90	−1.00 CSmeanC − 0.59 CsoptmeanA
1125	35.88	−1.00 CSmeanC − 4.84 CSbestB
1150	19.93	1.00 CSmeanD − 15.28 CSmeanB
1175	37.38	1.00 CsoptbestB − 1.66 CsoptmeanA
1200	21.35	1.00 CsoptmeanD − 1.73 CsoptmeanB
1225	29.90	−1.00 CsoptmeanC + 0.50 CsoptbestB
1250	28.47	1.00 CsoptmeanD − 0.21 CsoptbestC − 0.98 CSmeanB

**Table 4:** Frequency of the scores from the four simulated optimized monomers in the selected consensus equations. The table also includes the mean volumes of the four binding sites in the 50 selected frames (in Å^3^).

Score	Monomer A	Monomer B	Monomer C	Monomer D	Total
Best	10	11	10	5	36
Mean	11	19	15	18	63
Total	21	30	25	23	99
Mean volume	491.2	884.8	648.2	591.0	653.8

**Table 5:** Average values for the EF1% metrics and volumes of the binding pockets for optimized and non-optimized frames as computed by subdividing the MD run into five segments of 250 ns (the volume averages are expressed in Å^3^).

Time (ns)	Non-Optimized Frames		Optimized Frames	
	EF1%	Volume Average	EF1%	Volume Average
25–250	14.61	380.6	17.13	556.1
275–500	19.90	428.1	20.40	630.4
525–750	24.48	503.1	23.79	655.2
775–1000	19.14	525.8	25.48	711.8
1025–1250	26.75	512.52	29.70	715.5
Averages	21.38	470.0	23.29	653.8

**Table 6:** Best performances as derived from consensus analysis of multiple non-optimized and optimized frames (in parenthesis the EF1% means as obtained by averaging the 20 generated models).

EF1%	All Monomers	Monomer A	Monomer B	Monomer C	Monomer D	Best Scores	Mean Scores
Non-optimized	47.84 (37.07)	29.90 (24.17)	41.86 (29.00)	44.85 (36.36)	44.85 (30.65)	35.88 (13.85)	44.84 (26.54)
Optimized	49.83 (38.62)	44.85 (36.13)	29.90 (26.48)	41.86 (26.61)	44.85 (36.63)	35.88 (25.12)	38.94 (29.90)

**Table 7:** Comparison of the here reached performances with that obtained by the best model of the previous study [13].

Metric	Best Model from Previous Study [13]	Multiple Non-Optimized Frames	Multiple Optimized Frames
EF1%	67.11	47.84	49.83
% active in top 1%	67.11%	80%	90%
MCC	0.66	0.49	0.64
Sensitivity	0.66	0.50	0.65
Accuracy	0.99	0.99	0.99

## Data Availability

Data is contained within the article.

## Supplementary Materials

The following is available online https://www.mdpi.com/article/10.3390/ijms23147558/s1.

## Abbreviations

APBS	Adaptive Poisson–Boltzmann Solver
CS	Chemical Score
CSopt	Optimized Chemical Score
EF	Enrichment Factor
EFO	Enrichment Factor Optimization
MCC	Matthews correlation coefficient
MD	Molecular Dynamics
MLPInS	Molecular Lipophilicity Potential Interaction Score
PLP	Piecewise Linear Potential
PS	Pacman Score
TRPM8	Transient receptor potential cation channel subfamily M (melastatin) 8
VS	Virtual Screening
